# The Potential Role of Cannabinoids in Modulating Serotonergic Signaling by Their Influence on Tryptophan Metabolism

**DOI:** 10.3390/ph3082647

**Published:** 2010-08-13

**Authors:** Marcel Jenny, Sebastian Schröcksnadel, Florian Überall, Dietmar Fuchs

**Affiliations:** 1 Division of Medical Biochemistry, Biocenter, Innsbruck Medical University, 6020 Innsbruck, Austria; 2 Division of Biological Chemistry, Biocenter, Innsbruck Medical University, 6020 Innsbruck, Austria

**Keywords:** tryptophan, indoleamine-2,3-dioxygenase, Δ9-tetrahydrocannabinol, cannabidiol

## Abstract

Phytocannabinoids present in *Cannabis* plants are well known to exert potent anti-inflammatory and immunomodulatory effects. Previously, we have demonstrated that the psychoactive Δ9-tetrahydrocannabinol (THC) and the non-psychotropic cannabidiol (CBD) modulate mitogen-induced Th1-type immune responses in peripheral blood mononuclear cells (PBMC). The suppressive effect of both cannabinoids on mitogen-induced tryptophan degradation mediated by indoleamine-2,3-dioxygenase (IDO), suggests an additional mechanism by which antidepressive effects of cannabinoids might be linked to the serotonergic system. Here, we will review the role of tryptophan metabolism in the course of cell mediated immune responses and the relevance of cannabinoids in serotonergic signaling. We conclude that in particular the non-psychotropic CBD might be useful for the treatment of mood disorders in patients with inflammatory diseases, since this cannabinoid seems to be safe and its effects on activation-induced tryptophan degradation by CBD were more potent as compared to THC.

## 1. Introduction

Cannabis species such as *Cannabis sativa* (L.) or *Cannabis indica* (Lam.) produce more than 60 cannabinoids, of which the psychoactive Δ9-tetrahydrocannabinol (THC) and the non-psychotropic cannabidiol (CBD) are the most abundant components of these herbs. Cannabinoids exert their pharmacological effects by activating specific G-protein coupled cannabinoid receptors (CB1/2), present on central and peripheral nerves, but also on immune cells, which generated growing interest in evaluating the potential of cannabinoids as anti-inflammatory and immunomodulatory agents [1,2]. THC was found to exhibit potent anti-inflammatory and immunosuppressive effects on macrophages, natural killer (NK) cell activity and T lymphocytes, including e.g., suppression of mitogen-stimulated proliferation, interleukin (IL)-2 production, T cell-dependent antibody responses and secretion of pro-inflammatory cytokine tumor necrosis factor (TNF-α) [3,4,5,6]. Furthermore, THC was also reported to regulate the Th1-/Th2-type cytokine balance in activated human T cells polarizing the immune response towards a Th2 phenotype, which is considered to be beneficial in various diseases associated with inflammation [7]. However, besides reported inhibitory effects of cannabinoids on cells of the immune system, there are also studies demonstrating stimulatory activities. On the one hand, both THC and CBD were shown to decrease TNF-α production in human NK cells and peripheral blood mononuclear cells (PBMC), whereas THC was demonstrated to increase TNF-α production in human monocytes [8,9]. Similarly, treatment of human PBMC with low doses of THC or CBD, comparable to plasma levels detectable after smoking marijuana (10-100 ng/mL), was demonstrated to stimulate interferon (IFN)-γ production, while higher concentrations of these cannabinoids (5-20 µg/mL) efficiently suppressed formation of this cytokine [9]. These contradictory findings are suggested to be based on a biphasic response relative to the cannabinoid ligand concentration applied, since most of reports showing stimulatory capacities were reported at lower doses, in the nanomolar concentration range, whereas inhibitory activities of cannabinoids were found in the micromolar concentration range [10,11]. These concentration dependent effects of cannabinoids could be demonstrated for Th1- as well as Th2-type cytokines [12]. In the past decade, much attention has been focused on the mechanism of action of CBD, which was also shown to exert potent anti-inflammatory and immunomodulatory effects [13,14]. In addition, CBD has been reported to exhibit anticonvulsive [15],antianxiety [16], and antipyschotic activity [17,18]. 

**Figure 1:**
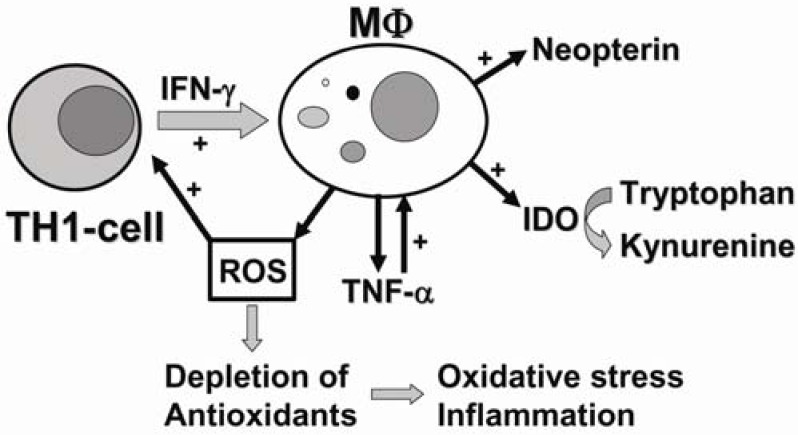
In the course of the adaptive immune response, activated T-helper (Th)1-type immune cells produce cytokines such as interleukin-2 or interferon-γ (IFN-γ). Pro-inflammatory cytokine IFN-γ stimulates several antiproliferative effector functions in monocyte-derived macrophages (MΦ), such as production of reactive oxygen species (ROS) and the activity of indoleamine-2,3-dioxygenase (IDO) and GTP-cyclohydrolase I, which are responsible for the conversion of tryptophan to kynurenine and the production of neopterin, respectively. Formation of ROS trigger redox-sensitive signal transduction cascades including the production of tumor necrosis factor-α (TNF-α), which enhances sensitivity of macrophages to pro-inflammatory IFN-γ. Continuous release of ROS may overwhelm the antioxidant capacity of cells, whereby oxidative stress and inflammation develop.

Previously, we have demonstrated that both cannabinoids, THC and CBD, modulate mitogen-induced Th1-type immune responses in PBMC in a biphasic manner [19]. In this study, we could show that mitogen induced production of neopterin, a marker of cellular immunity, was dose-dependently suppressed upon treatment of PBMC with THC or CBD. While pretreatment of PBMC with nanomolar doses of THC or CBD induced an increase of phytohemagglutinin (PHA)-stimulated IFN-γ secretion, application of micromolar doses efficiently suppressed activation induced production of this pro-inflammatory cytokine. The biphasic effect of THC and CBD could also be observed on mitogen-induced degradation of the essential amino acid tryptophan, catalyzed by indoleamine-2,3-dioxygenase (IDO), which constitutes an important mechanism of the adaptive immune defence system (Figure 1).

**Figure 2:**
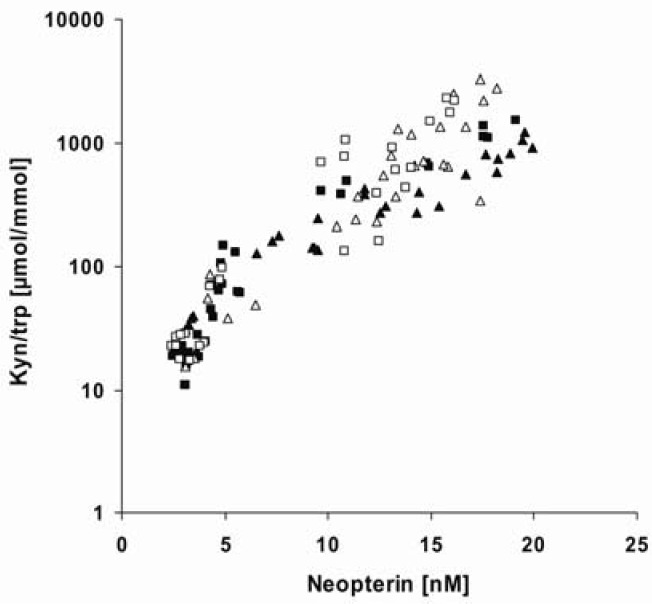
Associations between neopterin production and kynurenine to tryptophan ratio (Kyn/trp; shown in log scale) in phytohemagglutinin (PHA; open symbols) and concanavalin A(Con A; solid symbols)-stimulated peripheral blood mononuclear cells (PBMC) pretreated with Δ9-Tetrahydrocannabinol (THC; triangles) or cannabidiol (CBD; squares) at doses of 1-10 µg/mL. Neopterin production was positively associated with Kyn/trp in PHA-stimulated PBMC pretreated with THC (rs = 0.868; p < 0.001; n = 30) or CBD (rs = 0.909; p < 0.001; n = 30), as well as in Con A-stimulated PBMC pretreated with THC (rs = 0.962; p < 0.001; n = 26) or CBD (rs = 0.909; p < 0.001; n = 30). Tryptophan and kynurenine concentrations were measured by high-performance liquid chromatography (HPLC) [20], and concentrations of neopterin by a commercially available enzyme linked immunosorbent assay (BRAHMS, Hennigsdorf, Germany). Non-parametric correlations were calculated using Spearman’s rank test.

Again, nanomolar concentrations of THC and CBD enhanced mitogen-induced enzyme activity of IDO in PBMC, which could be shown to be dependent on CB1- and CB2-receptor activation. In contrast, micromolar doses of these cannabinoids strongly down-regulated mitogen-stimulated tryptophan degradation, independent of either cannabinoid receptor. Notably, CBD was about 2-4 times more active than THC to suppress IDO enzyme activity. Interestingly, tryptophan degradation mediated by spontaneous IDO activity in unstimulated cells, was significantly diminished by THC or CBD, already at nanomolar doses of 0.1 µg/mL. The simultaneous inhibition of mitogen-stimulated tryptophan degradation and neopterin formation, suggests a suppressive effect of THC and CBD on activated T-cells and production of IFN-γ, which could be confirmed in PHA-stimulated PBMC on the level of IFN-γ mRNA expression and secretion. As shown in Figure 2, neopterin production strongly correlated to kynurenine to tryptophan ratios (kyn/trp), as a measure of IDO enzyme activity, in mitogen stimulated PBMC pretreated with THC or CBD within a concentration range of 1-10 µg/mL. However, in THP-1 monocytes the suppressive effect of THC and CBD on lipopolysaccharide (LPS)-induced tryptophan degradation was even more effective, implying that inhibition of IDO activity is not only achieved via suppression of T cell-derived IFN-γ but also by a direct effect on stimulated monocytes.

In addition to our results on the anti-inflammatory/immunosuppressive effects of THC and CBD in mitogen stimulated PBMC, we suggest an involvement of these cannabinoids in the modulation of serotonergic signaling by their capacity to increase the availability of circulating tryptophan, the precursor necessary for the biosynthesis of the neurotransmitter 5-hydroxytryptamine (5-HT; serotonin). The alteration of serotonergic activity has been shown in many pathological processes, especially in neuropsychiatric disorders, such as depression and mood disorders [21,22]. Thus, the compensation of tryptophan degradation might be an important mechanism, by which THC and CBD may improve mood disturbances and quality of life, especially in diseases associated with inflammation.

## 2. The Role of Tryptophan Metabolism in Diseases Associated with Inflammation

The essential amino acid tryptophan is required for protein synthesis and serves as a precursor of a large number of metabolites, which play an important role in human nutrition and metabolism. About 95% of dietary tryptophan is metabolized by the kynurenine pathway, the primary route of tryptophan degradation in mammalian cells, leading to formation of metabolites such as nicotinamide adenine dinucleotide (NAD+), kynuramines, kynurenic acid, quiloninic acid and picolinic acid [23]. One of the biological functions of the kynurenine pathway is the regulation of plasma levels of tryptophan by the clearance of excess circulating tryptophan. While most of ingested tryptophan is metabolized by the glutarate pathway, yielding CO_2_ and ATP, most metabolites of the kynurenine pathway exert their biological activities especially in the nervous and immune systems [24,25]. The biosynthesis of kynurenines and NAD+ is initialized by the predominantly hepatic tryptophan 2,3-dioxygenase (TDO) and IDO, which is expressed in various peripheral cells such as macrophages and cells of the central nervous system [26]. While TDO accepts only tryptophan as a substrate and is activated primarily by glucocorticoids, IDO metabolizes also 5- hydroxytryptophan (5-HTB), serotonin and tryptamin, and is induced by Th1-type cytokine IFN-γ and some other pro-inflammatory stimuli [27,28,29,30]. Consequently, the second important pathway of tryptophan metabolism is the serotonin pathway, in which tryptophan is hydroxylated by the rate limiting enzyme tryptophan-5-hydroxylase (TPH) and decarboxylated by aromatic acid decarboxylase (AADC) yielding the neurotransmitter serotonin, which appears to be strongly involved in the pathogenesis of mood disorders and depression [31].

During inflammatory processes, IDO triggered tryptophan degradation is an important mechanism of the anti-microbial and anti-proliferative response of the human immune defence system [32,33]. In the course of the adaptive immune response against intracellular pathogens such as viruses, parasites and bacteria, Th1-type immune cells are activated and release large amounts of cytokines, such as IL-2 and IFN-γ [34,35]. Pro-inflammatory cytokine IFN-γ is probably the most important multiplier of anti-microbial and anti-tumoral host defence mediating a variety of physiological and cellular responses e.g., induction of high amounts of anti-microbial and cytocidal reactive oxygen species (ROS) by macrophages and other cells, directed to inhibit the growth and proliferation of cells and pathogens [36,37]. Moreover, ROS are also capable to interfere with various redox-sensitive intracellular signal-transduction cascades involving, e.g., activation of nuclear factor-κB (NF-κB), which in turn potentiates the production of pro-inflammatory cytokines such as TNF-α and IFN-γ [35,38,39,40]. Consequently, accumulated ROS further amplify Th1-type immune responses, and thereby act as positive regulators in addition to pro-inflammatory Th1-type cytokines (Figure 1).

As mentioned above, in human macrophages T-cell derived IFN-γ involves also stimulation of the enzyme activity of IDO, which converts tryptophan into N-formylkynurenine that is subsequently deformylated to kynurenine [41]. The resulting limited availability of tryptophan in the circulation inhibits protein synthesis and thus the growth of intracellular microorganisms such as bacteria, parasites and viruses, but also of highly proliferating tumor cells [29,42,43,44,45]. Furthermore, the depletion of tryptophan by IDO suppresses T-cell responsiveness, which may be important in tolerance induction [46,47]. *In vitro* studies have shown that interferons are the most effective stimuli for the activation of IDO, and IFN-γ has been shown to exert the strongest potential to induce IDO enzyme activity in human monocytes and macrophages [48,49]. In parallel to tryptophan degradation, neopterin concentrations also increase upon stimulation of GTP-Cyclohydrolase I by IFN-γ, representing another marker for the activation of the T cell-macrophage axis (Figure 1) [41,50].

Significant associations between blood levels of IFN-γ, neopterin and accelerated tryptophan degradation have been found in various diseases associated with an activated cell-mediated immune response, such as human immunodeficiency virus (HIV) infection, malignancy and autoimmune syndromes [41,51,52,53]. Moreover, the decreased availability of tryptophan in such conditions was found to be associated with reduced quality of life and an increased risk of depression, e.g., in patients with cancer or undergoing treatment with pro-inflammatory cytokines [54,55,56,57]. Thereby, activation of IDO enzyme activity could represent a link between the immunological network and the pathogenesis of depression, as the availability of tryptophan limits serotonin biosynthesis [58,59].

## 3. Involvement of Cannabinoid Receptor Signaling in Depression and Serotonergic Signaling

Cannabis consumption may induce anxiolytic, euphoric and rewarding effects and causes very complex subjective experiences in humans such as mood elevation, enhanced sensitivity to external stimuli and relaxation [60,61]. However, also psychotic symptoms such as panic attacks, anxiety and mood disturbances have been reported after chronic cannabis use [60]. These observations may be attributed to dose-dependent and biphasic effects of cannabinoid receptor agonists in several animal models of anxiety, where e.g., low doses of THC or nabilone induced anxiolytic effects and high doses of THC, or the cannabinoid receptor agonist HU210, produced anxiogenic-like responses [62,63,64,65,66,67].

Cannabinoids and the endocannbinoid system are well known to be involved in the regulation of mood and depression and the modulation of the serotonergic system in the central nervous system [68,69,70,71]. Experiments in CB1 knockout mice revealed, that stimulation of cannabinoid receptors is linked to a reduction in depressive behaviours implicating that cannabinoids may exhibit antidepressant activity [72]. Bambico and Gobbi suggested, that the CB1 agonist anandamide and inhibitors of the endocannabinoid hydrolyzing enzyme fatty acid amide hydrolase (FAAH) may represent possible antidepressant targets, since both were shown to enhance central serotonergic and noradrenergic transmission [73]. Direct evidence for the involvement of cannabinoids in depression was provided in animal studies using agonists and antagonists of cannabinoid receptors, although the interactions are very complex, which leads to controversial interpretations [74]. While mouse behavioral studies revealed an antidepressant effect of the CB1 receptor antagonist SR 141716A (rimonabant) [75,76], CB1 receptor agonists such as arachidonyl-2-chloroethylamide or HU-210 have also been shown to exert an antidepressant effect in the forced swim assay for mice and rat [77,78]. The involvement of the endocannabinoid system in depression has been linked to the serotonergic system via the activation of serotonin receptors 5-HT_1_, 5-HT_2_, 5-HT_3_, and the enhanced firing activity of serotonergic and noradrenerdic neurons after treatment with URB597, an inhibitor of FAAH [68,69]. Accordingly, URB597 showed antidepressant-like effects in a rat model of chronic mild stress [79]. Conflicting results were obtained on the influence of cannabinoids on serotonin turnover in rodents. The cannabinoid receptor agonist WIN55212-2 and the endocannabinoid anandamide were shown to inhibit dopamine and 5-HT uptake into rat neocortical synaptosomes [80]. Likewise, in the rat hippocampus, THC treatment was reported to reduce serotonin turnover and the CB1 receptor antagonist rimonabant was shown to stimulate serotonin release from the prefrontal cortex [81,82]. In contrast, experiments analyzing the content of serotonin in different brain regions of adult rats revealed a marked increase of serotonin in the frontal cortex of rats chronically treated with THC [74]. Additionally, the CB1 receptor antagonists rimonabant and AM251 were both found to decrease the firing rate of dorsal raphe nucleus 5-HT neurons in rat brain slices, while administration of the CB1 receptor agonist HU-210 has been shown to activate the hypothalamic-pituitary-adrenal (HPA) axis in Sprague-Dawley rats indirectly through an increase in serotonergic and noradrenergic neurotransmission [83,84]. Furthermore, the anxiolytic-like effects of CBD, tested in the elevated plus maze and the Vogel conflict test in rats, have been shown to be mediated by direct activation of 5-HT_1A_ receptors [85,86].

Evidence of an involvement of cannabinoid signaling in the development of depression in humans, was unfortunately recognized after the approval and clinical use of rimonabant (Acomplia^®^) in the management of obesity [87]. In contrast to the above mentioned antidepressant effects of this CB1 antagonist in rodents, some patients taking Acomplia as an anti-obesity drug suffered from serious adverse effects. Acomplia treatment was accompanied by an increased incidence of depression, anxiety and suicidality, leading finally to the removal of Acomplia from the market [88,89,90,91]. Recently, in rodent models of mood disorders, chronic rimonabant treatment was also shown to produce depressive-like symptoms, which were paralleled by a decrease in frontal cortex serotonin levels and an increase of pro-inflammatory cytokines IFN-γ and TNF-α [92].

Although clinical trials on the influence of cannabinoids in affective disorders yielded contradictory results, many patients continue to report benefits from its use in primary or secondary depressive syndromes. However, this assumption has to be proven in further clinical studies [93,94,95,96].

## 4. Conclusions

Results of our *in vitro* study demonstrated that THC and CBD interfere with immunological pathways stimulated by pro-inflammatory Th1-type cytokine IFN-γ, which further emphasizes their well known anti-inflammatory capacity [19]. The suppressive effect of THC and CBD on cytokine-induced tryptophan degradation may constitute an additional mechanism by which antidepressive effects of cannabinoids might be linked to the serotonergic system. Disturbed balance of serotonin levels is an important risk factor for depressive mood, which is also a common symptom in the later course of chronic disorders such as cancer, infections and autoimmune syndromes [54,57,97]. Many patients with chronic inflammatory diseases show accelerated tryptophan degradation and an increased susceptibility for depressive mood, implicating a role of cytokine-induced IDO enzyme activity in psychiatric diseases [41,98]. Additionally, several studies showed, that mood is negatively influenced by depletion of tryptophan [99,100]. Since tryptophan is essential for the biosynthesis of serotonin, the decreased availability of tryptophan during inflammatory conditions as a result of degradation by IDO, may negatively affect the biosynthesis of this neurotransmitter [57]. Furthermore, findings of our study regarding the effects of THC and CBD to increase tryptophan concentrations in unstimulated PBMC in the nanomolar concentration range, may give some explanation for the observed well-being after smoking marijuana. Results on the cannabinoid receptor-dependent enhancement of tryptophan degradation by nanomolar concentrations of THC and CBD in activated PBMC might represent an adverse effect, which should be considered when evaluating the *in vivo* effects of cannabinoids on the emotional state during inflammatory diseases.

Therapeutic application of *Cannabis* and accordingly the psychoactive THC is limited by the occurrence of side effects, such as sedation, dysphoria, unpleasant subjective feelings and the potential to cause addiction and tolerance [101]. In contrast, CBD, which has been shown to exhibit largely analogue anti-inflammatory effects, is devoid of adverse psychoactive properties and has a safe profile in humans [102,103]. Our results, on the suppression of activation-induced tryptophan degradation by THC and CBD in cells of the immune system is all the more interesting, since the effect of CBD on this pathway was 2-4 times more potent in comparison to THC. Therefore, the non-psychotropic CBD is an attractive compound to improve mood disturbances and quality of life by its influence on tryptophan, and consequently serotonin metabolism, especially in diseases associated with inflammation. It is always difficult or sometimes even impossible to extrapolate *in vitro* results to the situation *in vivo*. The effect of low cannabinoid doses to increase tryptophan degradation in PBMC *in vitro* may relate to short-time effects of cannabinoids *in vivo*, whereas the effect of high doses to suppress tryptophan degradation *in vitro* may represent the situation of a chronic long-lasting effect of cannabinoid exposure at even lower concentrations, which are achievable in the circulation of living organisms. Further behavioural studies are needed to confirm our assumption of an effect of cannabinoids on tryptophan and serotonin metabolism. Although our findings were obtained *in vitro*, they might have manifold consequences also for the *in vivo* situation since serotonin, in addition to behaviour and depression, regulates numerous further biological processes such as pain, cardiovascular, gastrointestinal, genitourinary and reproductive function, breathing and pulmonary hypertension [104].
